# LBF-MI: Limited Boolean Functions and Mutual Information to Infer a Gene Regulatory Network from Time-Series Gene Expression Data

**DOI:** 10.3390/genes15121530

**Published:** 2024-11-27

**Authors:** Shohag Barman, Fahmid Al Farid, Hira Lal Gope, Md. Ferdous Bin Hafiz, Niaz Ashraf Khan, Sabbir Ahmad, Sarina Mansor

**Affiliations:** 1 Department of Computer Science and Engineering, Bangabandhu Sheikh Mujibur Rahman Science and Technology University, Pirojpur 8500, Bangladesh; 2 Faculty of Engineering, Multimedia University, Cyberjaya 63000, Selangor, Malaysia; fahmid.farid@mmu.edu.my; 3 Faculty of Agricultural Engineering and Technology, Sylhet Agricultural University, Sylhet 3100, Bangladesh; hlgope@sau.ac.bd; 4 Department of Computer Science and Engineering, Southeast University, Dhaka 1208, Bangladesh; ferdousbinhafiz@gmail.com; 5 Department of Computer Science and Engineering, University of Liberal Arts Bangladesh, Dhaka 1207, Bangladesh; niaz.ashraf@ulab.edu.bd; 6 Department of Computer Science and Engineering, University of Chittagong, Chittagong 4331, Bangladesh; sabbir3337@gmail.com

**Keywords:** network inference, gene regulatory network, mutual information, Boolean functions

## Abstract

Background: In the realm of system biology, it is a challenging endeavor to infer a gene regulatory network from time-series gene expression data. Numerous Boolean network inference techniques have emerged for reconstructing a gene regulatory network from a time-series gene expression dataset. However, most of these techniques pose scalability concerns given their capability to consider only two to three regulatory genes over a specific target gene. Methods: To overcome this limitation, a novel inference method, LBF-MI, has been proposed in this research. This two-phase method utilizes limited Boolean functions and multivariate mutual information to reconstruct a Boolean gene regulatory network from time-series gene expression data. Initially, Boolean functions are applied to determine the optimum solutions. In case of failure, multivariate mutual information is applied to obtain the optimum solutions. Results: This research conducted a performance-comparison experiment between LBF-MI and three other methods: mutual information-based Boolean network inference, context likelihood relatedness, and relevance network. When examined on artificial as well as real-time-series gene expression data, the outcomes exhibited that the proposed LBF-MI method outperformed mutual information-based Boolean network inference, context likelihood relatedness, and relevance network on artificial datasets, and two real *Escherichia coli* datasets (*E. coli* gene regulatory network, and SOS response of *E. coli* regulatory network). Conclusions: LBF-MI’s superior performance in gene regulatory network inference enables researchers to uncover the regulatory mechanisms and cellular behaviors of various organisms.

## 1. Introduction

A directed graph, containing a set of molecular components such as genes, proteins, and mRNA, and their genetic interactions, is utilized to represent a gene regulatory network (GRN). Network inference means discovering genetic interactions from time-series gene expression data. In system biology, reconstructing a GRN from time-series data is significantly intricate. Network inference facilitates identifying complex regulatory relationships among genes and thus exposes the regulatory mechanisms of an organism. It also assists researchers in understanding the regulatory relationships between genes and diseases [1,2]. The rapid progress in high-throughput technology has significantly increased the availability of extensive gene expression data, enabling researchers to reconstruct gene regulatory networks [3].

Numerous methods have been proposed for inferring gene regulatory networks (GRNs) based on various computational models, including Bayesian networks [4], differential equations [5], and Boolean networks [6]. Among them, the Boolean network is one of the most popular computational models because it is simple to model gene states as binary variables. Moreover, the Boolean network is well suited for modeling large-scale gene regulatory networks and has been ubiquitously used in previous studies [7,8,9,10]. In the Boolean network, a gene’s state is represented as either 0 (OFF) or 1 (ON), and the states are updated using Boolean functions. However, scalability remains a significant issue in many Boolean network models. For example, three widely-known techniques—the reverse engineering algorithm (REVEAL) [11], Best-Fit [12], and the Bayesian inference approach for Boolean networks (BIBN) [13]—restrict the number of regulatory genes to two or three when reconstructing GRNs from time-series gene expression data. To address this limitation, mutual information has been employed to reconstruct Boolean networks from such data. For instance, the relevance network (RelNet) proposed by Butte et al. [14] calculates mutual information between gene pairs and retains interactions above a specific threshold. The context likelihood of relatedness (CLR) [15] method improves upon this by introducing an adaptive background correction to reduce false correlations in the relevance network. Another method, the algorithm for reconstruction of accurate cellular networks (ARACNE), calculates mutual information between gene pairs and applies the data preprocessing inequality (DPI) to eliminate the weakest edges within gene triplets (I(X, Y, Z)) to construct the GRN [16]. Although these approaches demonstrated notable performances, they are limited by their focus on pairwise relationships, preventing them from fully representing multivariate mutual information. In another study, the **mutual information-based Boolean network inference (MIBNI)** [7] was proposed for Boolean network inference, which can calculate approximate multivariate mutual information. It has two subroutines: **MIFS** and **SWAP**. First, **MIFS** uses mutual information to select k important features. Then, **SWAP** is employed to swap regulatory genes between the set of selected and unselected regulatory genes to improve dynamic accuracy. If the consistency of the gene-wise dynamic is improved, the set of regulatory genes is considered the final predicted set of regulatory genes for a target gene. MIBNI has a local optimum problem. Although intriguing performances were achieved in each study, they were not able to represent multivariate mutual information due to considering only pairwise relations.

To overcome the aforementioned limitations, this study presents a novel approach called LBF-MI, which combines limited Boolean functions with multivariate mutual information to infer gene regulatory networks from time-series gene expression data. The LBF-MI method operates in two phases. In the first phase, it systematically explores all possible combinations of regulatory genes for a target gene and identifies the function that minimizes errors to zero. If the optimal solution is not found, the method proceeds to examine all combinations of regulatory gene sets and selects the one that maximizes mutual information, which signifies the optimal solution. This process is carried out independently for each target gene. The final results from all target genes are then aggregated to form the inferred Boolean network. To evaluate the performance of LBF-MI, it was compared against three widely recognized methods, MIBNI, RelNet, and CLR. The experimental results demonstrated that LBF-MI significantly outperformed both methods in terms of accuracy on both artificial and real gene expression datasets.

The remainder of the paper is organized as follows: Section 2 narrates the materials and method including the Boolean network inference problem, structural performance metrics, random Boolean network, random Boolean network generation, generation of time-series gene expression dataset or Boolean trajectories from random Boolean networks, multivariate mutual information, and workflow of the proposed method. Section 3 concentrates on the proposed approach of this research. Section 4 portrays the outcomes and comparative analysis of this research. Section 5 provides the conclusion and future research direction.

## 2. Materials and Methods

### 2.1. Boolean Network Inference Problem

The Boolean network inference problem involves inferring a set of interactions between target genes and regulatory genes, as well as determining the update functions from time-series gene expression data. The performance of the inference can be evaluated by comparing the Boolean trajectories of the inferred network with those of the target network. Let v(t) represent the Boolean trajectories of the observed gene expression data, and v′(t) represent the estimated gene expression data. Gene-wise dynamic consistency can be defined by comparing the similarities between v(t) and v′(t), as follows:

(1) $$GDC\left(v,\;v^{\prime }\right)=\frac{\sum_{t=l+1}^{S}I(v\left(t\right)=v^{\prime }\left(t\right))}{S-l}$$

where S is the total number of time steps, l is the time lag, and $I\left(.\right)$ is a indicator function that returns 1 if the condition is true and otherwise return 0. In this study, l is set to 1, and comaprisrons start at t=2. Gene regulation is a complex process that does not occur instantaneously in biological systems. A time lag of 1 accounts for the delay between the activation or repression of a regulator (such as a transcription factor) and the subsequent change in the target gene’s expression level. This delay reflects the intricate and realistic time required for transcription, translation, and other regulatory mechanisms to unfold in molecular biology. Gene-wise dynamics consistency quantitatively contributes to inference accuracy, as the greater the consistency between predicted Boolean trajectories and experimental data, the more accurate the inference of gene regulatory relationships and network structure.

### 2.2. Structural Performance Matrices

If the gold standard network is known, the structural performance between the target network, G(V,A), and the predicted network, G(V′,A′) can be evaluated. The F-Score is computed using Equation (1).

(2) $$F\text{-}\mathrm{Score}=\frac{2\mathrm{pr}}{p+r}$$

Here p and r denote precision and recall are denoted by p and r respectively. Equation (2) represents Precision as the ratio of accurately inferred connections over the aggregate number of predictions.

(3) $$\;p=\frac{\mathrm{TP}}{\mathrm{TP}+\mathrm{FP}}\;$$

Here, TP (true positive) represents the number of connections correctly identified by the LBF-MI algorithm that also exist in the target network, while FP (false positive) indicates the number of connections predicted by the LBF-MI algorithm that do not exist in the target network. Recall is calculated using Equation (3) as the ratio of correctly identified connections to the total number of actual connections.

(4) $$\;r=\frac{\mathrm{TP}}{\mathrm{TP}+\mathrm{FN}}\;$$

Here FN (false negative) denotes connections that exist in the target network but cannot be inferred by the LBF-MI algorithm.

### 2.3. A Random Boolean Network

To represent gene regulation, in 1969 Kauffman introduced a Boolean network comprising a set of nodes $V=\{V_{1},V_{2},V_{3},\ldots ,\;V_{n}$} and a set of Boolean functions (to update the value of each node) $F=\left\{f_{1},f_{2},f_{3},\;\ldots ,\;f_{n}\right\}$ [6], wherein 0 and 1 represent the states of genes in a Boolean network, where 1 symbolizes the gene being “ON” or expressed, and 0 represents the gene’s state as “OFF” or not expressed. A state vector of all nodes represents the state of the Boolean network. In a random Boolean network, when connected by a directed link, nodes $v_{i}$ and $v_{j}$ can have any of the two regulatory relationships: positive (“activation”) or negative (“inhibition”). To determine the binary value of the variable $v_{i}\in \;V$ at time t+1, a Boolean function is applied on the values of other variables $v_{i1},v_{i2},\ldots v_{ik}$ which have a direct link to $v_{i}$ at t. Equation (5) exhibits this computation.

(5) $${\;v}_{i}\left(t+1\right){=f}_{i}\left(v_{i1}{,v}_{i2}{,\ldots v}_{\mathrm{ik}}\right)\left(t\right)$$

After that, a synchronous update occurs on all the network variables.

### 2.4. Generation of Random Boolean Networks

In this study, the LBF-MI algorithm is thoroughly evaluated using both artificial and real-time-series gene expression datasets. To generate artificial datasets, the Barabási–Albert (BA) model was utilized to construct random Boolean networks. This model has recently gained significant attention as a prominent approach for modeling gene regulatory networks. Using the number of nodes (N) and a probability (p) that any two nodes are connected, the following constraints were applied to generate the random network:

- Minimum two nodes are required for connectivity;
- It is essential for each node to possess at least one incoming edge and at least one outgoing edge.

**Figure 1:**
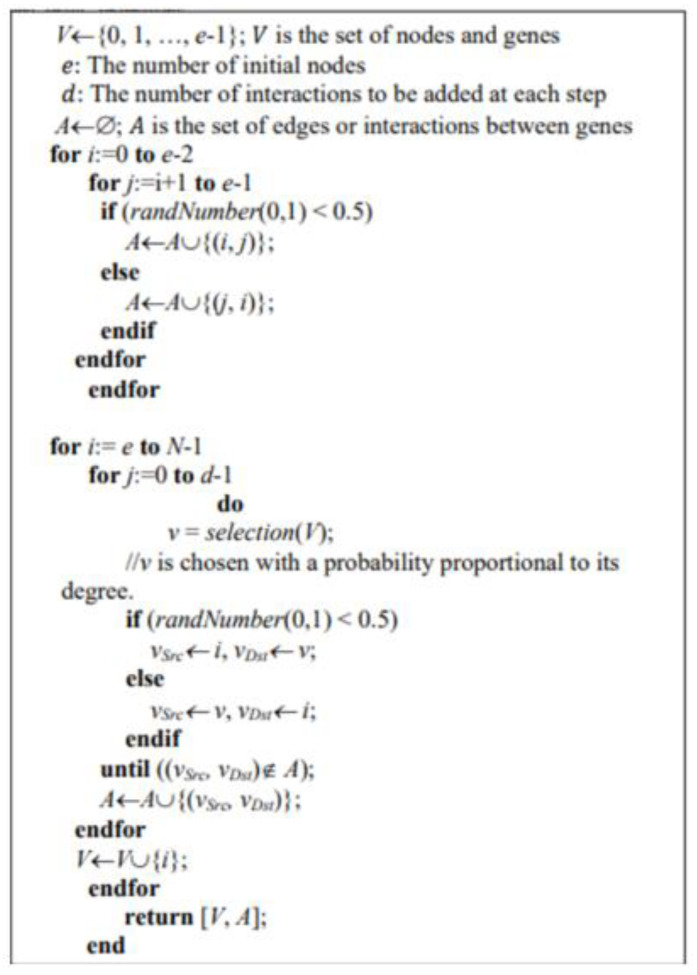
Pseudo-code for the BA model.

Figure 1 displays the pseudo-code for the BA model, which includes the following parameters in each iteration: the total number of nodes (N), the number of nodes in the initial seed network (e), and the number of edges added at each iteration (d).

In the BA model, we mainly use three key parameters: N (the desired number of nodes), e (the number of initial nodes), and d (the number of interactions to be added at each step). The choice of e is critical as it determines how quickly the network evolves into a scale-free structure, with the initial regulatory molecules (such as transcription factors) acting as the starting nodes. The parameter d controls how many interactions a new node (e.g., a gene or protein) forms, with higher values leading to a denser network and lower values resulting in a sparser one. This reflects the preferential attachment principle, where new nodes are more likely to connect to existing nodes with higher degrees. N represents the final network size, corresponding to the scale of the biological system being modeled. Smaller networks, like gene regulatory pathways, may have hundreds of nodes, while larger networks, such as entire regulatory systems, may involve thousands. Together, these parameters enable the model to capture the growth dynamics and connectivity patterns in real biological networks.

### 2.5. Generation of Time-Series Gene Expression Dataset or Boolean Trajectories from Random Boolean Networks

The BA model, proposed by Barbasi et al. [17], is frequently employed by the researchers to generate a scale-free random Boolean network [6]. In this study, 10 groups of random Boolean networks were created, each with varying network sizes ranging from |V| = 10 to 100, with edges |A| = 2.|V|. To generate time-series gene expression datasets or Boolean trajectories for various random Boolean network sizes, the following steps were executed:

1. Consider a random initial state in which each value represents the state of a gene.
2. Then, formulate a random update function, which considers any of the two Boolean functions: AND or OR while updating each gene.
3. Calculate the next state.
4. Repeat step 3 until a steady state is found.

The sequence of states generated by this iterative process forms the Boolean trajectory or Boolean time-series gene expression dataset.

### 2.6. Multivariate Mutual Informational

Our proposed approach selects a regulatory variable (gene) or a set of regulatory variables (genes) that is based on some concepts of information theory. First, the entropy $H\left(X\right)$ of a discrete random variable (gene) X is defined by Equation (6).

(6) $$\;H\left(X\right)=-\underset{x\in X}{\sum }p\left(x\right)\mathrm{logp}\left(x\right)$$

In addition, the joint entropy $H\left(X,Y\right)$ of two discrete random variables X and Y with a joint probability distribution $p\left(x,y\right)$ is defined by Equation (7).

(7) $$H\left(X,Y\right)=-\underset{x\in X}{\sum }\underset{y\in Y}{\sum }p\left(x,y\right)\mathrm{logp}\left(x,y\right)$$

Moreover, the entropy of a set of variables X, Y, Z  measures the uncertainty with their joint distribution. If X, Y, Z are discrete random variables, the joint entropy $H\left(X,Y,Z\right)$ can be denoted by Equation (8).

(8) $$H\left(X,Y,Z\right)=-\underset{x\in X}{\sum }\underset{y\in Y}{\sum }\underset{z\in Z}{\sum }p\left(x,y,z\right)\mathrm{logp}\left(x,y,z\right)$$

Here $p\left(x,\;y,\;z\right)$ is the joint probability mass function of $\left(X,Y,Z\right)$.

We used the mutual information of two discrete variables using Equation (9).

(9) I(X; Y)=H(X)+H(Y) − H(X, Y)

Finally, Equation (10) calculates the multivariate mutual information between target gene T and the set of regulatory genes $S=\left\{X,\;Y,\;Z\right\}$.

(10) I(T;X;T;Z)=H(T)+H(X,Y,Z) − H(T,X,Y,Z)

The larger the mutual information I, the more dependent the regulatory genes are on the target gene.

### 2.7. Workflow of the Proposed Method

**Figure 2:**
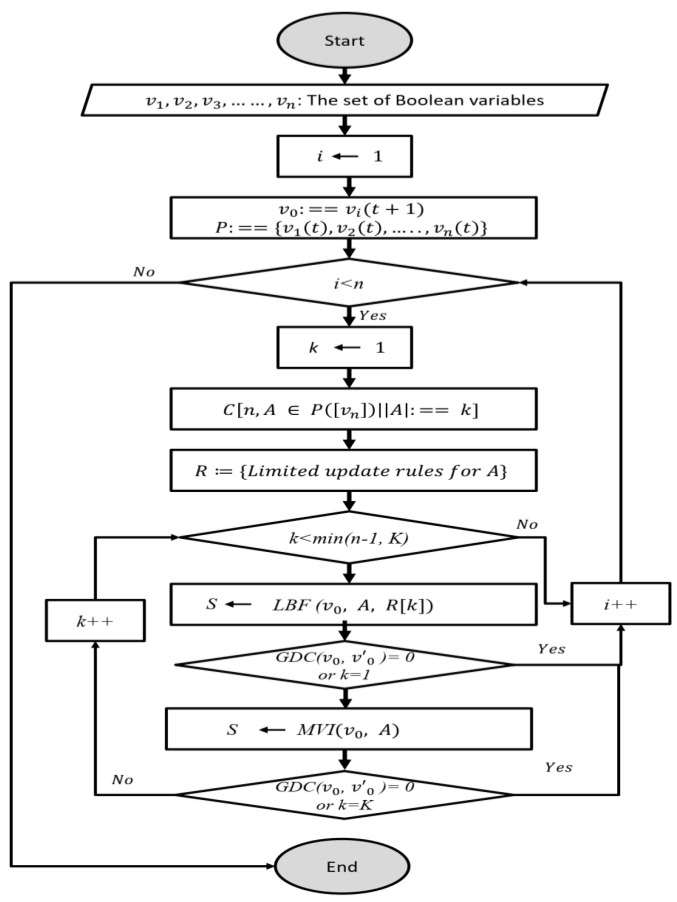
Workflow of LBF-MI.

LBF-MI runs limited Boolean functions and multivariate mutual information independently to select k regulatory genes for each target gene in each iteration, denoted as i. Given, a target gene $v_{0}$, a set of candidate variables P= { $v_{1},\;v_{2}\ldots \ldots \ldots .\;v_{n}\},$ and two parameters: k, which represents a single regulatory gene, and K, which denotes the maximum number of regulatory genes. Additionally, $\left|A\right|=k$ represents a subset of candidate regulatory genes. For each iteration i, we generate combinatorial sets of regulatory genes $C\left(n,\;k\right)$ based on the value of k, where n denotes the total number of candidate genes. Initially, we formulate update rules for the parameter k. LBF-MI first employs LBF, which exhaustively searches for a regulatory gene when k=1 that best approximates $GDC\left(v_{0},\;v_{0}^{\prime }\right)=0$, where the regulatory gene will be inferred for a target gene. If $GDC\left(v_{0},\;v_{0}^{\prime }\right)=0$ is not achieved, LBF-MI then employs the multivariate mutual information between the target gene and regulatory genes, $MVI\left(v_{0},\;A\right).$ The process is repeated by increasing the value of k  until an optimal regulatory gene set is found or until k  reaches the parameter K. The workflow of LBF-MI is illustrated in Figure 2.

## 3. Proposed Method

This research proposes a novel method, LBF-MI, to infer a gene regulatory network from time-series gene expression data.

**Figure 3:**
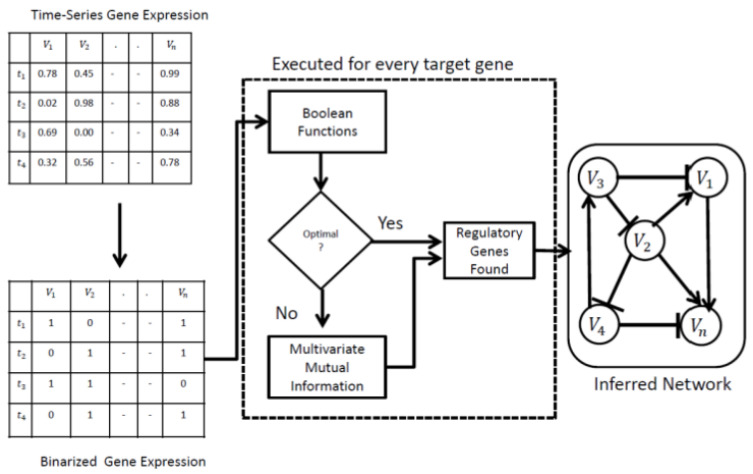
Overall framework of LBF-MI.

The overall framework of the proposed method is illustrated in Figure 3.

A real-time-series gene expression dataset is used as input and transformed into a Boolean gene expression dataset through the K-means algorithm [18]. The K-means algorithm begins by randomly selecting two initial data points as centroids, one for each cluster, corresponding to the gene expression data of each target gene. Next, each data point is assigned to the cluster whose centroid is closest, measured by Euclidean distance. Once all points have been assigned to their respective clusters, the centroids are updated by calculating the mean of the points within each cluster. This process of reassigning points to clusters and recalculating centroids is repeated until the clusters reach a stable configuration, signaling convergence. Once the clustering is complete, the centroids of the two clusters are compared. Each centroid represents the average gene expression value for its cluster, which is used as a threshold. If the centroid of Cluster 0 is greater than that of Cluster 1, the data points in Cluster 0 are assigned a binary value of 1, while those in Cluster 1 are assigned a value of 0. The proposed LBF-MI works in two steps.

(a) In the first phase, LBF-MI is employed to find the optimum solutions for a target gene. Optimum solutions are simply the regulatory genes for a target gene, achieved when the gene-wise dynamics between the target gene and the regulatory gene reach 1 (see Materials and Methods section for more details). Consider a target gene, $v_{0},$ and a regulatory gene, $v_{1}.$ In this study, a one-time gap is used between a target gene and a regulatory gene. For a given target gene, initially, an exhaustive search was conducted for its regulatory interactions, considering two Boolean functions: $v^{\prime }\left(t+1\right)=v_{1}\left(t\right)$ and $v^{\prime }\left(t+1\right)=∼v_{1}\left(t\right),\;$ and the optimal fitting function was identified that approximates the gene-wise dynamics consistency to 1. Gene-wise dynamics consistency is the similarity between the predicted Boolean time-series data $v^{\prime }\left(t\right)$ and the observed data $v_{0}\left(t\right).$ The optimal fitting function refers to one of the two Boolean functions mentioned above that maximizes gene-wise dynamics consistency. If there is no difference between the Boolean time-series data $v^{\prime }\left(t\right)\;$ and $v_{0}\left(t\right)$, an optimal regulatory gene is obtained. Otherwise, step 2 is executed.
(b) In the second phase, this research conducts an exhaustive search for regulatory gene tuples using multivariate mutual information. The multivariate mutual information measures how much information is shared among the target gene and regulatory genes $I\left(v_{0};v_{1},\ldots ,v_{n}\right)$ by considering both their entropies and their joint entropy (see Materials and Methods section). First, a value of k equal to 1 is selected, specifying the computation of mutual information between a target gene and a regulatory gene. Subsequently, the value of k is incremented by 1 iteratively until k reaches the limit of K.  Finally, the best tuple with the highest mutual information score was selected. It is worth mentioning that step 1 and step 2 are executed independently for every target gene. All results are combined to build a final random Boolean network.

## 4. Results

In this study, two types of gene expression datasets were used: artificial and real gene expression datasets. Initially, the artificial gene expression dataset was used to evaluate the proposed method, and its performance was compared with three well-known methods, MIBNI, RelNet, and context likelihood relatedness (CLR). The proposed method was subsequently applied to two real gene expression datasets: *E. coli* gene regulatory network and regulatory network of the SOS response of *E. coli*.

### 4.1. Performance on Artificial Gene Expression Dataset

**Figure 4:**
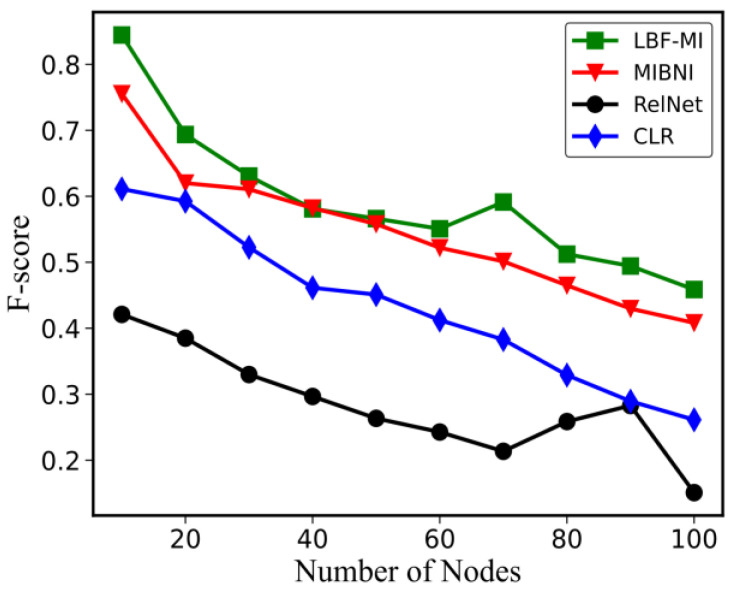
Comparisons of F-score between LBF-MI and other methods in BA random networks.

To evaluate the proposed method, ten groups of randomly generated networks were constructed using the BA model with different network sizes (|V| = 10, 20, 30, …, 100) and |E| = 2 ⋯ |V|. For every network size, ten networks were generated, resulting in a total of 100 networks. Following that, 100 time-series gene expression datasets were generated, and the maximum time-step (T) was configured to |V| + 10 for the BA model. The LBF-MI method was compared with three well-known methods, MIBNI, RelNet, and CLR, over 100 artificial gene expression datasets for structural accuracy. The X-axis of Figure 4 represents the average F-score values for each group, while the Y-axis shows the network size for each group, ranging from 10 to 100 nodes. We further compared LBF-MI with two well-known methods, GABNI [8] and NNBNI [9], and found that LBF-MI may not achieve a better F-score than these methods. However, the running time complexity of LBF-MI is significantly lower than that of the other two methods. Figure 4 demonstrates that the average F-score is higher for smaller networks and decreases as the network size increases. This trend can be attributed to the fact that the number of incoming links generally reflects the complexity of the inference problem for each network. From Figure 4, it is clear that, among the three methods, LBF-MI outperforms MIBNI, RelNet, and CLR in terms of F-score. MIBNI was set with a parameter *K* of 10, while, for a fair comparison with LBF-MI, K was set to 4. We evaluated the effect of the k parameter by calculating the average F-scores across 5 random Boolean networks (See Table 1). We observed that when k=5, the F-scores dropped significantly, as a larger K results in more regulatory genes failing to regulate a target gene. As a result, we chose to set the parameter at k=4.

### 4.2. Performance on the Real Gene Expression Dataset

To validate the performance of LBF-MI, it was experimented on two biological networks: *Escherichia coli* (*E. coli*) gene regulatory network and SOS response of the *E. coli* gene regulatory network.

#### 4.2.1. Case Study 1: *E. coli* Gene Regulatory Network

**Figure 5:**
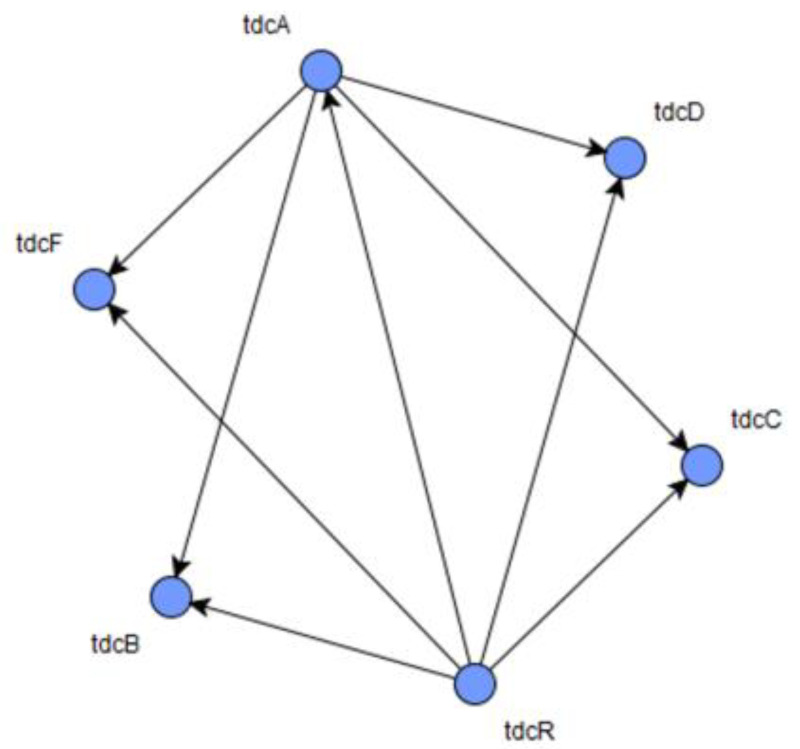
Gold standard structure of *E. coli* network consisting of 6 nodes and 9 interactions.

The performance of LBF-MI was evaluated using a time-series gene expression dataset derived from an *E. coli* gene regulatory network, comprising six nodes and nine interactions, generated with the GeneNetWeaver (GNW) tool [19]. Initially, the dataset was produced in real-valued form from the *E. coli* network and later converted into a Boolean format through a discretization method based on the K-means clustering algorithm. The reference structure of the *E. coli* gene regulatory network is illustrated in Figure 5. LBF-MI successfully reconstructed all nine interactions within the network, whereas MIBNI identified four interactions, and both RelNet and CLR accurately identified only two interactions each.

In Table 2, the proposed approach has achieved higher values for F-score (0.6000), Precision (0.4285), and Recall (1.000) compared to MIBNI, RelNet and CLR. This implies that the proposed method has significantly outperformed MIBNI, RelNet and CLR methods in terms of structural accuracy.

#### 4.2.2. Case Study 2: Regulatory Network of the SOS Response of *E. coli*

A Boolean network model for SOS response of the *E. coli* consisting of 6 nodes and 10 interactions (Self-loop is not considered) was presented in a previous study by Shao et al. [20], which also provided a time-series gene expression dataset of Boolean values represented in Table 3.

**Figure 6:**
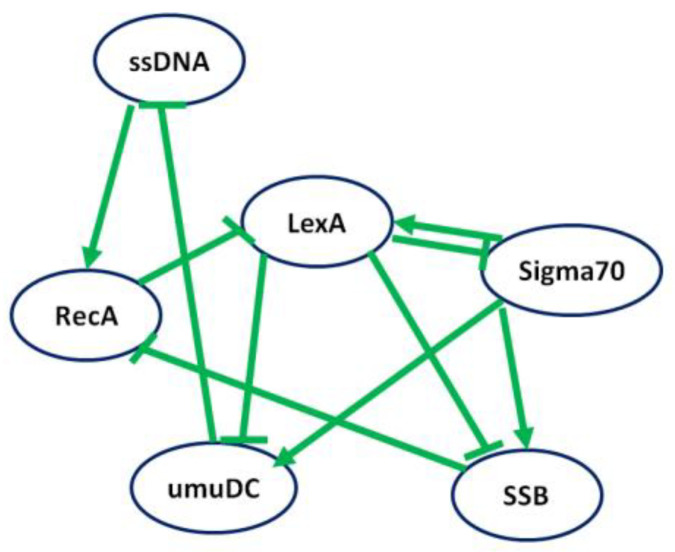
Gold standard structure of SOS response *E. coli* regulatory network consisting of 6 nodes and 10 interactions.

Figure 6 illustrates the interactions between nodes, with a positive interaction depicted by an arrow connecting two genes, while negative interactions are represented by lines ending with a bar. The maximum time step of the time-series gene expression data was set to 8.

**Figure 7:**
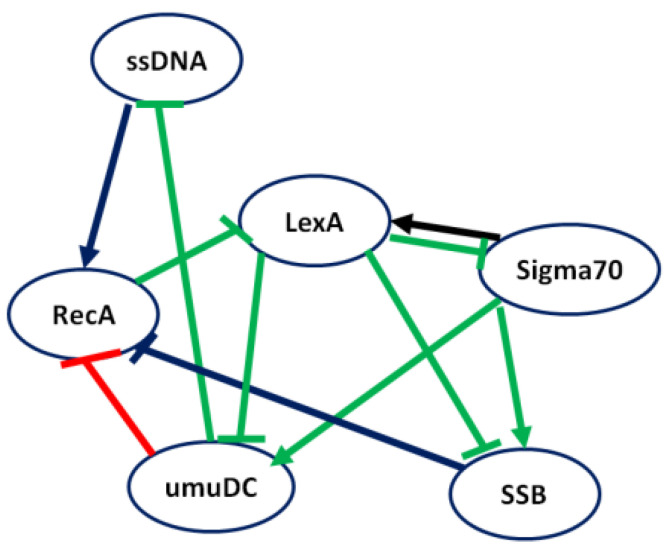
Inference performance of LBF-MI for SOS response *E. coli* regulatory network.

Figure 7 depicts the network inferred by LBF-MI, where green, red, and blue edges represent true positive, false positive, and false negative predictions, respectively. As shown, LBF-MI accurately inferred 7 out of 10 interactions. In comparison, MIBNI, RelNet, and CLR correctly identified 6, 6, and 5 interactions, respectively.

Table 4 presents the evaluation of inference performance, including F-score, Precision, and Recall, for LBF-MI, MIBNI, RelNet, and CLR on the regulatory network of the SOS response in *E. coli*. LBF-MI has achieved an F-score of 0.7000, Precision of 0.6666, and Recall of 0.5263, respectively. These values are higher than those obtained by MIBNI, RelNet, and CLR. LBF-MI exhibited significantly better performance than MIBNI, RelNet, and CLR in terms of structural accuracy.

### 4.3. Computation Time Comparisons

**Figure 8:**
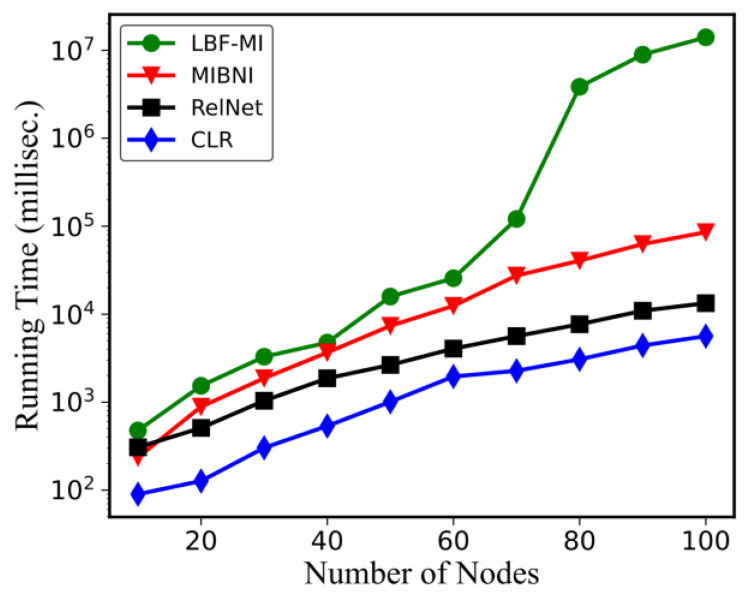
Comparison of the running time among LBF-MI, MIBNI, RelNet, and CLR.

Figure 8 illustrates the comparative analysis of the running times for LBF-MI, MIBNI, RelNet, and CLR across 10 BA random networks. The simulations were performed on a PC with an Intel Core i5 1.60 GHz processor and 8 GB of memory, utilizing a single core. The Y-axis represents the average logarithmic running times for the four algorithms. Among these methods, CLR exhibited the fastest performance. Conversely, LBF-MI recorded the slowest time due to its exploration of a larger problem space. This suggests that our approach achieves high-quality solutions at the expense of longer search times. Time units are measured in milliseconds.

## 5. Discussion and Conclusions

Reconstructing a gene regulatory network from time-series gene expression data is a challenging task in systems biology. Researchers have developed numerous inference methods for network inference. However, most of these methods are not scalable because of limiting the regulatory genes from one to three. To obtain better scalability, this research proposes a novel method named LBF-MI which has constrained the number of regulatory genes by four, enhancing its scalability compared to other existing methods. While some other well-known methods inferred gene regulatory networks through pairwise mutual information, LBF-MI, in contrast, inferred networks using multivariate mutual information, a common approach in systems biology. Pairwise mutual information measures the relationship between the target gene and regulatory gene, capturing direct dependencies but missing complex interactions involving multiple regulatory genes for a target gene. In contrast, multivariate mutual information evaluates the joint dependency of multiple genes simultaneously, allowing it to capture higher-order interactions common in biological systems, where gene regulation often involves multiple factors, not just pairs of genes. Moreover, multivariate mutual information lies in its ability to reduce the occurrence of false positives by considering the joint dependency of multiple genes, allowing it to capture more accurate relationships and minimize spurious correlations that might arise in pairwise mutual information. Furthermore, MIBNI uses approximated multivariate mutual information to reduce computational complexity. However, this approach may result in local optimum issues, especially when handling more complex regulatory relationships. On the other hand, LBF-MI employs exact multivariate mutual information, which offers a more accurate understanding of the relationships between target genes and regulatory genes in a gene regulatory network, though it comes with higher computational demands. A performance evaluation of the proposed method was conducted on artificial datasets and two real networks, namely the *E. coli* gene regulatory network and the SOS response of *E. coli* regulatory network. LBF-MI demonstrated superior accuracy compared to other state-of-the-art methods in both artificial and real gene expression datasets. The scalability of LBF-MI is particularly valuable for genome-scale network inference (GRN reconstruction), where it can efficiently manage large, complex datasets. This makes it especially useful for inferring GRNs in large model organisms like *Arabidopsis thaliana* and *Mus musculus*, which involve processing extensive genomic and transcriptomic data. By enabling the streamlined reconstruction of regulatory networks across whole genomes or large systems, LBF-MI has the potential to accelerate breakthroughs in functional genomics, systems biology, and precision medicine. A limitation of LBF-MI is that it has the slowest running times. A parallel implementation can be conducted to reduce the search time in the forthcoming research. Moreover, a neural network can be utilized alongside multivariate mutual information to infer a gene regulatory network more accurately from time-series data.

## Figures and Tables

**Table 1:** Effect of k values on F-score values.

*k*	1	2	3	4	5	6	7	8	9
F-Score	0.6600	0.7972	0.8047	0.8190	0.7291	0.7291	0.6810	0.6810	0.6810

**Table 2:** The F-score, precision and recall for LBF-MI, RelNet, and CLR over the regulatory network of the SOS response in *E. coli*.

Methods	F-Score	Precision	Recall
LBF-MI	0.6000	0.4285	1
MIBNI	0.2424	0.1666	0.4444
RelNet	0.1999	0.1888	0.2222
CLR	0.1904	0.1666	0.2222

**Table 3:** The F-score, precision and recall for LBF-MI, MIBNI, RelNet, and CLR over the regulatory network of the SOS response in *E. coli*.

Time	ssDNA	RecA	LexA	Sigma 70	UmuD C	SSB
1	1	0	1	0	0	0
2	1	1	1	0	0	0
3	1	1	0	0	0	0
4	1	1	0	1	0	0
5	1	1	0	1	1	1
6	0	0	0	1	1	1
7	0	0	1	1	1	1
8	0	0	1	0	0	0

**Table 4:** The F-score, precision, and recall for LBF-MI, MIBNI, RelNet, and CLR over the regulatory network of the SOS response in *E. coli*.

Methods	F-Score	Precision	Recall
LBF-MI	0.7000	0.8750	0.7000
MIBNI	0.3414	0.2916	0.4117
RelNet	0.6666	0.7500	0.6000
CLR	0.5263	0.5555	0.5000

## Data Availability

The original contributions presented in this study are included in the article. Further inquiries can be directed to the corresponding author.
